# Plasmacytoid Dendritic Cells Suppress HIV-1 Replication but Contribute to HIV-1 Induced Immunopathogenesis in Humanized Mice

**DOI:** 10.1371/journal.ppat.1004291

**Published:** 2014-07-31

**Authors:** Guangming Li, Menglan Cheng, Jun-ichi Nunoya, Liang Cheng, Haitao Guo, Haisheng Yu, Yong-jun Liu, Lishan Su, Liguo Zhang

**Affiliations:** 1 Key Lab of Infection and Immunity, Institute of Biophysics, Chinese Academy of Sciences, Beijing, China; 2 Lineberger Comprehensive Cancer Center, University of North Carolina at Chapel Hill, Chapel Hill, North Carolina, United States of America; 3 Baylor Institute for Immunology Research, Baylor Research Institute, Dallas, Texas, United States of America; 4 Department of Microbiology and Immunology, University of North Carolina at Chapel Hill, Chapel Hill, North Carolina, United States of America; Emory University, United States of America

## Abstract

The role of plasmacytoid dendritic cells (pDC) in human immunodeficiency virus type 1 (HIV-1) infection and pathogenesis remains unclear. HIV-1 infection in the humanized mouse model leads to persistent HIV-1 infection and immunopathogenesis, including type I interferons (IFN-I) induction, immune-activation and depletion of human leukocytes, including CD4 T cells. We developed a monoclonal antibody that specifically depletes human pDC in all lymphoid organs in humanized mice. When pDC were depleted prior to HIV-1 infection, the induction of IFN-I and interferon-stimulated genes (ISGs) were abolished during acute HIV-1 infection with either a highly pathogenic CCR5/CXCR4-dual tropic HIV-1 or a standard CCR5-tropic HIV-1 isolate. Consistent with the anti-viral role of IFN-I, HIV-1 replication was significantly up-regulated in pDC-depleted mice. Interestingly, the cell death induced by the highly pathogenic HIV-1 isolate was severely reduced in pDC-depleted mice. During chronic HIV-1 infection, depletion of pDC also severely reduced the induction of IFN-I and ISGs, associated with elevated HIV-1 replication. Surprisingly, HIV-1 induced depletion of human immune cells including T cells in lymphoid organs, but not the blood, was reduced in spite of the increased viral replication. The increased cell number in lymphoid organs was associated with a reduced level of HIV-induced cell death in human leukocytes including CD4 T cells. We conclude that pDC play opposing roles in suppressing HIV-1 replication and in promoting HIV-1 induced immunopathogenesis. These findings suggest that pDC-depletion and IFN-I blockade will provide novel strategies for treating those HIV-1 immune non-responsive patients with persistent immune activation despite effective anti-retrovirus treatment.

## Author Summary

Persistent expression of IFN-I is correlated with disease progression in HIV-1 infected humans or SIV-infected monkeys. Thus, persistent pDC activation has been implicated in contributing to AIDS pathogenesis. To define the role of pDC in HIV-1 infection and immunopathogenesis *in vivo*, we developed a monoclonal antibody that specifically and efficiently depletes human pDC in all lymphoid organs in humanized mice. We discover that pDC are the critical IFN-I producer cells in response to acute HIV-1 infection, because depletion of pDC completely abolished induction of IFN-I or ISG by HIV-1 infection, correlated with elevated level of HIV-1 replication. When pDC were depleted during chronic HIV-1 infection in humanized mice, pDC were still the major IFN-I producing cells *in vivo*, which contributed to HIV-1 suppression. Despite of higher level of viral replication in pDC-depleted mice, we found that HIV-induced depletion of human T cells and leukocytes was significantly reduced in lymphoid organs, correlated with reduced cell death induction by HIV-1 infection. Our findings demonstrate that pDC play two opposing roles in HIV-1 pathogenesis: they produce IFN-I to suppress HIV-1 replication and induce death of human immune cells to contribute to HIV-induced T cell depletion and immunopathogenesis.

## Introduction

Chronic immune activation induced by HIV-1 infection is highly correlated with CD4 T cell depletion and immunodeficiency [1], [2], [3]. The level of T cell activation (HLA-DR^+^CD38^+^CD8^+^ T cells) is correlated with disease progression independent of HIV-1 viral load and CD4^+^ T cell count [4]. It is also proposed that immune activation drives AIDS development in simian immunodeficiency virus (SIV) infected monkeys. In SIV-infected Asian monkeys (Rhesus macaques and pigtail macaques, e.g.) AIDS develops, associated with persistent immune activation and rapid CD4^+^ T-cell loss. In contrast, SIV infection of African monkeys (African Green monkeys and sooty mangabeys, e.g.) leads to no AIDS progression, correlated with only a transient and self-limiting immune activation despite similar levels of viral replication as pathogenic SIV infections [2], [5], [6]. In mice, repeated treatments with Toll like receptor (TLR)-9 [7] or TLR7 [8] ligands lead to AIDS-like immune dysregulation, correlated with immune activation and lymphoid organ destruction. In SIV-infected African green monkeys, treatment with lipopolysaccharide (LPS) results in CD4^+^ T-cell loss [9]. Finally, anti-inflammatory treatment with chloroquine [10] or hydroxychloroquine in combination with antivirals [11] inhibits immune activation in HIV-1 infected patients, correlated with elevated CD4^+^ T cells [11].

The mechanism by which HIV-1 infection leads to immune activation is not fully elucidated [2]. Several mechanisms have been proposed, including loss of gut tissue integrity and microbial products translocation [12] or persistent production of IFN-I [13], [14]. Sustained IFN-I production is correlated with HIV-1 induced immune activation and disease progression both in HIV-1 infected patients [15] and pathogenic SIV infected monkey models [16], [17], [18]. Although IFN-I inhibits HIV-1 replication in vitro [19], the high level IFN-I in HIV-1 patients is not correlated with viral control but is predictive of HIV-1 disease progression [20], [21]. IFN-I is induced during acute phase of SIV infection in both pathogenic and non-pathogenic hosts. However, the IFN-I induction is controlled during nonpathogenic persistent SIV infection, while the pathogenic SIV infection is featured by sustained IFN-I production during chronic infection, correlated with immune activation and AIDS development [17], [22], [23] [16].

Plasmacytoid dendritic cells (pDC) are the major IFN-I producing cells [24]. They preferentially express TLR7 and TLR9 in the endosome, sensing viral RNA and DNA respectively during infection. Upon viral infections and other stimulations, pDCs produce large amount of IFN-I and inflammatory cytokines. However, it is still not clear if pDCs are the major source of IFN-I during acute or chronic HIV-1 infection [25], and the role of pDC in HIV-1 replication or disease progression is not well defined. HIV-1 infection can stimulate pDCs to express TNF-Related Apoptosis-Inducing Ligand (TRAIL) [26], [27], [28]. However, the induction of CD4^+^ T-cell death by TRAIL expressing pDC remains controversial [29]. On the other hand, pDC are also reported to be decreased and functionally impaired in peripheral blood of HIV-1 infected individuals [30], [31], [32], [33]. The decline of IFN-I producing capability of pDC is correlated with opportunistic infection but not CD4 T-cell counts [31], [34], [35]. These reports highlight that pDC may play important but complex roles in HIV-1 infection and immunopathogenesis.

Humanized mice transplanted with human immune tissues or cells have been developed to study HIV-1 infection [36]. In the recent improved humanized mouse models, HIV-1 infection can be established by inoculating through intraperitoneal [37], [38], intravenous [39], or mucosal routes [38], [40]. HIV-1 infection results in persistent viral replication, CD4 depletion in peripheral blood and lymphoid organs. Importantly, HIV-1 infection results in T cell depletion, correlated with immune activation in lymphoid organs of humanized mice [41].

We and other groups have reported that functional human pDC are developed in lymphoid tissues in humanized mouse models [41], [42], [43]. Human pDC are rapidly activated by HIV-1 infection [41] and the level of pDC activation is reversely correlated with CD4^+^ T-cell numbers [41], which is consistent with the observation from HIV-1 infected patients [15], [20], [21] and SIV infected monkeys [16], [23]. To define the role of human pDC in HIV-1 replication and immunopathogenesis in vivo, we developed a monoclonal antibody that specifically and efficiently depletes human pDC in all lymphoid organs in humanized mice in vivo. Thus we were able to characterize the role of human pDC in HIV-1 infection and immunopathogenesis during acute and chronic phases of HIV-1 infection.

## Results

### HIV-1 infection, immune activation and HIV-1 disease progression in humanized mice

**Figure 1:**
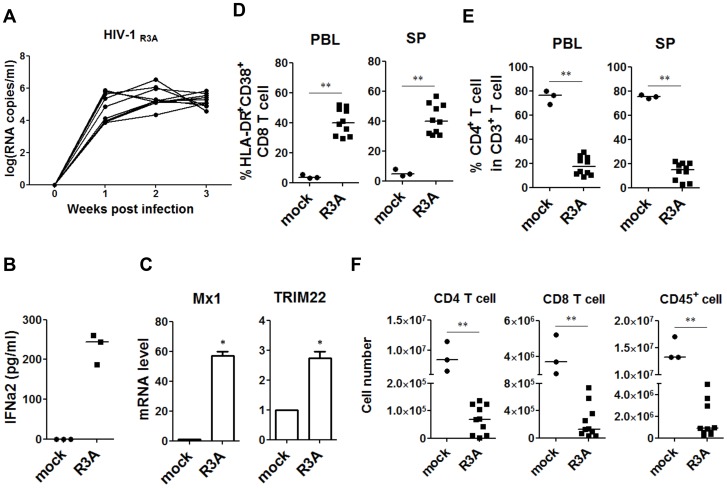
HIV-1 replication and pathogenesis in HIV-1-infected humanized mice. HIV-R3A replication and pathogenesis in humanized mice are summarized. (**A**) Plasma viral load of mice intravenously inoculated with 1 ng p24/mouse of R3A (*n* = 10). Viral RNA copy numbers were measured using real-time PCR quantification. (**B**) The production of IFN-a2 in plasma from uninfected (n = 3) and infected (n = 3) humanized mice measured by Luminex. (**C**) The relative level of Mx1 and TRIM22 gene expression in huCD45^+^ cell in spleen (*n* = 3) detected by real time PCR. (**D**) Summary data of the percentages of HLA-DR^+^CD38^+^ on CD8 T cells (CD3^+^CD4^−^CD8^+^) in peripheral blood and spleen measured by FACS. (**E**) Summary data of the percentages of CD4^+^ T cells of CD3^+^ cells. (**F**) Comparison of absolute CD4 T-cell, CD8 T-cell and huCD45^+^ cell numbers in spleen from uninfected controls (*n* = 3) and R3A-infected mice (*n* = 10). (B–F) Mice were analyzed at 8 days post infection. All bars in dot graphs indicate median value. Error bars indicate standard deviations (SD). * and ** indicate p<0.05 and p<0.01, respectively.

We have previously reported that human pDC are rapidly activated by HIV-1 infection [41] in humanized mice and the level of pDC activation is correlated with CD4^+^ T-cell depletion [41]. The pathogenic CCR5/CXCR4 dual-tropic HIV-R3A strain efficiently established infection in humanized mice (Figure 1A), associated with IFN-I induction and ISG expression (Figure 1B&C), increased HLA-DR^+^CD38^+^ CD8 T cells (Figure 1D), and CD4 T-cell depletion (Figure 1E). As in HIV-1 infected human patients, a decrease of total number of human leukocytes was induced by HIV-1 infection in humanized mice, as measured by cell numbers of human CD4, CD8 T cells and total CD45^+^ leukocytes in lymphoid organs (Figure 1F). As shown with chronic infection with JRCSF below, similar IFN-I induction, immune activation and depletion of human immune cells were observed in humanized mice (Figure S1A–E). We have reported that pDC frequency did not change during acute HIV-R3A infection [41]. During persistent JR-CSF infection, pDC percentage was also not significantly altered (data not shown). Thus, the humanized mouse model provides a relevant in vivo model for studying the role of pDC in HIV-1 infection and immunopathogenesis.

### Specific depletion of human pDC with a pDC-reactive monoclonal antibody

**Figure 2:**
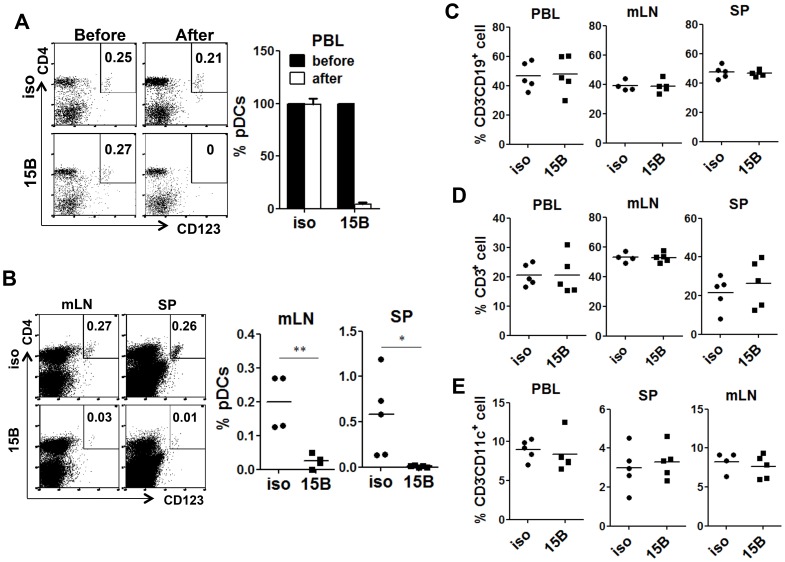
Specific depletion of human pDC in lymphoid organs in vivo with a human pDC-reactive monoclonal antibody. Humanized Mice were treated with either 15B or isotype control (iso) antibody for 3 times on days -5, -3, -1 prior to termination. Percentages of pDC (Lin^−^CD4^+^CD123^+^) in total human leukocytes (CD45^+^) are analyzed. (**A**) Representative FACS plots and summarized data show relative pDC frequencies before and after antibody treatment in the peripheral blood (n = 7). (**B**) Representative FACS plots and summarized data show pDC depletion by 15B in mesenteric lymph nodes (mLN) and spleen (SP, isotype n = 4; 15B n = 5). All bars in dot graphs indicate median value. Error bars indicate standard deviations (SD). * and ** indicate p<0.05 and p<0.01, respectively. (**C**) Summarized percentages of CD3^−^CD19^+^ B cells in different lymphoid tissues. (**D**) Summarized percentages of CD3^+^CD19^−^ T cells in different lymphoid tissues. (**E**) Summarized percentages of CD3^−^CD11c^+^ mDC cells in different lymphoid tissues. All bars in dot graphs indicate median value.

In order to delineate the role of human pDC in HIV-1 infection and pathogenesis in vivo, we developed and screened a number of pDC-reactive monoclonal antibodies (mAb) and identified an anti-BDCA2 (CD303) mAb (15B), which could specifically deplete human pDC in lymphoid organs in humanized mice. After 15B injection, pDC were specifically depleted in both peripheral blood (Figure 2A) and lymphoid organs (Figure 2B). Importantly, human T, B, myeloid dendritic cells and monocytes\macrophages were not perturbed by 15B mAb (Figure 2C–E and Figure S2&S3).

### Depletion of pDC prior to HIV-1 infection abolishes IFN-I induction and increases HIV-1 replication

**Figure 3:**
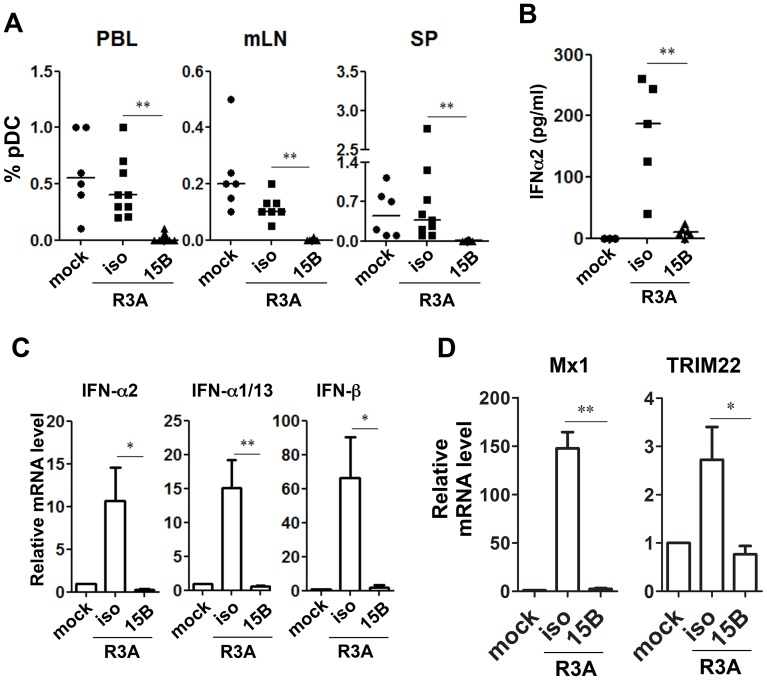
Pre-infection depletion of pDC abolishes IFN-I induction during acute HIV-1 infection in humanized mice. (**A**) Summarized data of pDC percentages in total human leukocytes (CD45^+^) from humanized mice are shown mice. Mice were treated with either 15B or isotype control (iso) antibody. After pDC depletion, mice were infected with HIV-R3A and terminated on 8 days post infection (dpi) for analysis. Mock infected mice, *n* = 6; isotype+R3A infected mice, *n* = 9; 15B+R3A infected mice, *n* = 12. (**B**) Plasma levels of IFN-α2 from mock, HIV-1 infected and 15B or isotype mAb treated mice were quantified by Luminex assays. Mock, *n* = 3; isotype+R3A, *n* = 5; 15B+R3A, *n* = 5. (**C**) The mRNA expression of major type I IFN genes in purified human cells (CD45^+^) from mouse spleens was measured by real-time PCR. (**D**) ISGs (Mx1 and TRIM22) expression in purified human cells (CD45^+^) from mouse spleens was measured by real-time PCR. Mock, *n* = 3; isotype+R3A, *n* = 5; 15B+R3A, *n* = 5. Mice were analyzed at 8 days post infection. Error bars in graphs indicate median value. Error bars indicate standard deviations (SD). * and ** indicate p<0.05 and p<0.01, respectively.

To test the role of pDC in early acute HIV-1 infection, we injected 15B and isotype control antibody into humanized mice on -5, -3 and -1 days before infection, and then infected them with HIV-R3A (a highly pathogenic dual-tropic HIV-1 strain, [44], [45]) on day 0. The infected mice were treated with 15B or control antibody two more times on 3 and 6 days post-infection (dpi). We found that pDC remained depleted in blood and lymphoid organs of the infected mice (Figure 3A), when terminated on 8 dpi. Interestingly, the induction of plasma IFN-I was completely blocked by pDC depletion in HIV-1 infected mice (Figure 3B). The suppressed expression of different subtypes of human IFN-I was also confirmed at RNA level by real time PCR (Figure 3C). In addition, the up-regulation of ISGs such as Mx1 and TRIM22 was also blocked (Figure 3D and data not shown). We confirmed similar blocking of IFN-I induction by pDC-depletion prior to infection with the CCR5-tropic JRCSF HIV-1 strain (data not shown). These data demonstrate that pDC are the critical IFN-I producing cells during early HIV-1 infection in humanized mice in vivo.

**Figure 4:**
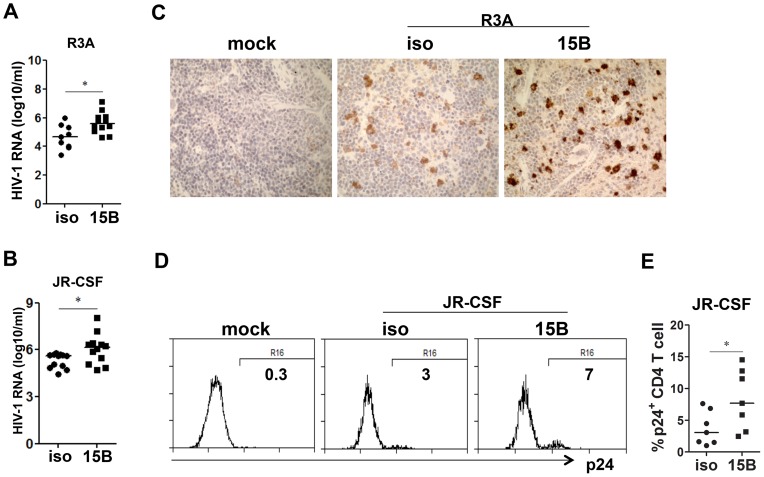
Pre-depletion of pDC leads to higher levels of HIV-1 replication. (**A**) Plasma HIV-1 RNA levels from HIV-R3A infected mice (genome copy#×log10/ml) are summarized. isotype+R3A, *n* = 9; 15B+R3A, *n* = 12. (**B**) Plasma HIV-1 RNA levels from JR-CSF infected mice at 2 wpi are summarized. isotype+JR-CSF, *n* = 12; 15B+JR-CSF, *n* = 12. (**C**) Immunohistochemistry staining for p24 positive cells in the spleen of mock or HIV-R3A infected mice. (**D**) Representative FACS plots for p24 positive CD4 T cells in the spleen of mock or JRCSF infected mice at 3 wpi. (**E**) Summarized data of D. isotype+JR-CSF, *n* = 7; 15B+JR-CSF, *n* = 7. Bars in dot graphs indicate median value. * indicates p<0.05.

Consistent with the antiviral activity of IFN-I, HIV-1 replication reached higher levels in pDC-depleted mice in vivo (Figure 4). The average plasma viral load was increased about 10-fold comparing with mice treated with isotype control antibody (Figure 4A, p<0.01). We repeated the pDC depletion experiment with the CCR5 tropic HIV-1 JR-CSF. Similar to HIV-R3A infection, JR-CSF replication was increased in pDC-depleted mice (about 5-fold, Figure 4B). The increase of viral replication was further confirmed in the spleen by immunohistochemistry (Figure 4C) or flow cytometry (Figure 4D&E) of HIV p24 protein positive cells. Therefore, pDC are the critical IFN-I producer cells in response to acute HIV-1 infection, and they are required to significantly inhibit early HIV-1 replication. The expression of HLA-DR and CD38 was increased in mice with pDC depletion (Figure S4), correlated with increased level of HIV-1 replication. These data suggest that the elevated level of HIV-1 replication in the absence of pDC can lead to upregulation of T-cell activation markers.

### Depletion of pDC reduces HIV-induced death of human CD4-negative leukocytes during acute HIV-R3A infection

**Figure 5:**
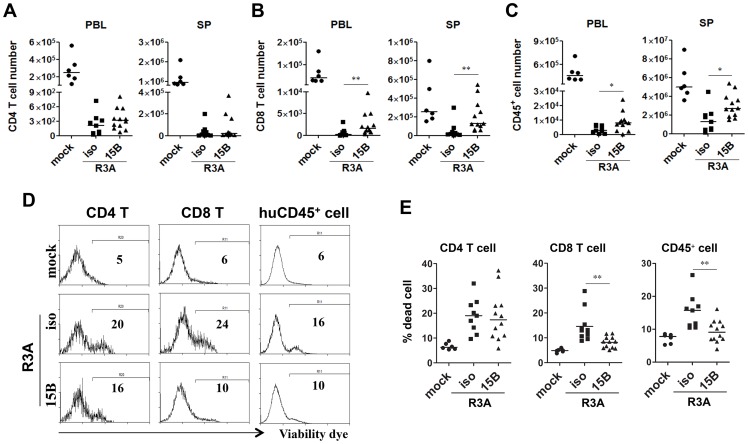
Pre-depletion of pDC reduces HIV-R3A induced death of human leukocytes. (**A**) CD4 T cell (CD3^+^CD8^−^) counts in peripheral blood (PBL) and spleens (SP) of mock or HIV-R3A infected mice. (**B**) CD8 T cell (CD3^+^CD4^−^CD8^+^) counts in PBL and SP. (**C**) Human CD45^+^ leukocyte counts in PBL and SP. (**D**) Representative histograms show percentages of dead CD4 T cells, CD8 T cells and huCD45^+^ cells in spleens of mice infected with mock or R3A at 8 dpi. (**E**) Summarized data of D. mock, *n* = 6; isotype+R3A, *n* = 9; 15B+R3A, *n* = 12. Bars in all graphs indicate median value. * and ** indicate p<0.05 and p<0.01, respectively.

As shown in Figure 1, HIV-R3A infection leads to rapid immunopathogenesis including depletion of human total leukocytes and CD8 T cells, as well as CD4 T cells. Despite the increased HIV-R3A viral replication in pDC-depleted mice, the absolute numbers of human CD4^+^ T cells in blood and spleen were comparable to those of control mice (Figure 5A). More surprisingly, CD8^+^ T cells as well as total human CD45^+^ leukocytes were partly preserved in blood and spleen in pDC-depleted mice (Figure 5B&C). Consistently, this is correlated with decreased levels of cell death of CD45 or CD8 T cells (Figure 5D&E). The similar cell death induction of CD4 T cells in both groups of mice may be due to the highly fusogenic activity of the HIV-R3A Env [45], [46]. The elevated HIV-R3A replication in pDC-depleted mice, combined with its highly pathogenic direct killing activity, may contribute to the observed CD4 T cell death induction and depletion.

### pDC remain as important IFN-I producer cells and inhibit viral replication during chronic HIV-1 infection in humanized mice

**Figure 6:**
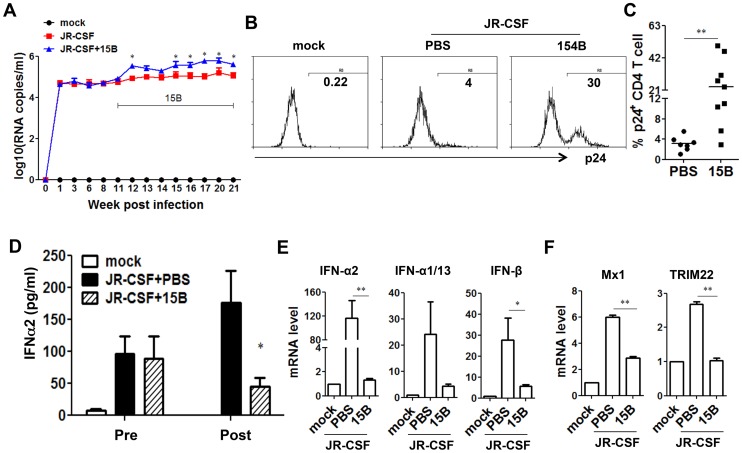
Depletion of pDC during chronic HIV-1 infection increases HIV-1 replication associated with reduced IFN-I expression. Humanized mice were infected HIV-JRCSF and were treated weekly with 15B or control at 11 weeks post-infection and terminated at 21 weeks post-infection (mock, *n* = 6; JR-CSF+control, *n* = 9; JR-CSF+15B, *n* = 9). (**A**) Plasma HIV-1 RNA levels (genome copy#×log10/ml) at each time point were analyzed by real-time PCR. (**B**) Representative FACS histograms and summarized data (**C**) show percentages of HIV p24-positive CD4 T cells (CD3^+^CD8^−^) in the spleen. (**D**) The production of IFNα2 in the plasma, from mock infected (n = 4), JR-CSF+PBS (n = 5) and JR-CSF+15B (n = 5) at either 11 wpi (pre-) or 21 wpi (post-), was measured by Luminex. (**E**) The mRNA levels of IFN-I genes or ISGs Mx1 and TRIM22 (**F**) in purified human CD45^+^ cells from spleens (n = 5). All bars in dot graphs indicate median value. Error bars indicate standard deviations (SD). * and ** indicate p<0.05 and p<0.01, respectively. Relative ISGs expression in human CD45^+^ cells or in CD8 T cells (CD3^+^CD4^−^CD8^+^) in spleens are summarized in Figure S5.

During early phase of JR-CSF infection, the decrease of CD4^+^ T cells and other human leukocytes was not significant (Figure S1). To define the role of pDC in HIV-1 replication and immunopathogenesis during chronic HIV-1 infection, we performed pDC depletion during chronic HIV-JRCSF infection. Humanized mice were infected with JR-CSF for 11 weeks, and then 15B was applied to deplete pDC for additional 10 weeks. In agreement with the data from acute infection, we observed a significant increase in plasma viremia (Figure 6A). The percentage of HIV-1 infected cells (HIV-1 p24 positive) was also significantly increased (Figure 6B&C). Interestingly, plasma IFN-α2 decreased significantly in the pDC-depleted mice (by 70%, Figure 6D). The mRNA induction of different IFN-I subtypes (Figure 6E) and ISGs (Figure 6F) in human leukocytes in spleens was almost completely suppressed by real-time PCR and cDNA array (Figure S5). Thus, pDC are still a major source of IFN-I, and contribute to suppressing HIV-1 chronic infection in humanized mice.

### Contribution of pDC to HIV-1 induced human leukocyte depletion in lymphoid organs during chronic infection

**Figure 7:**
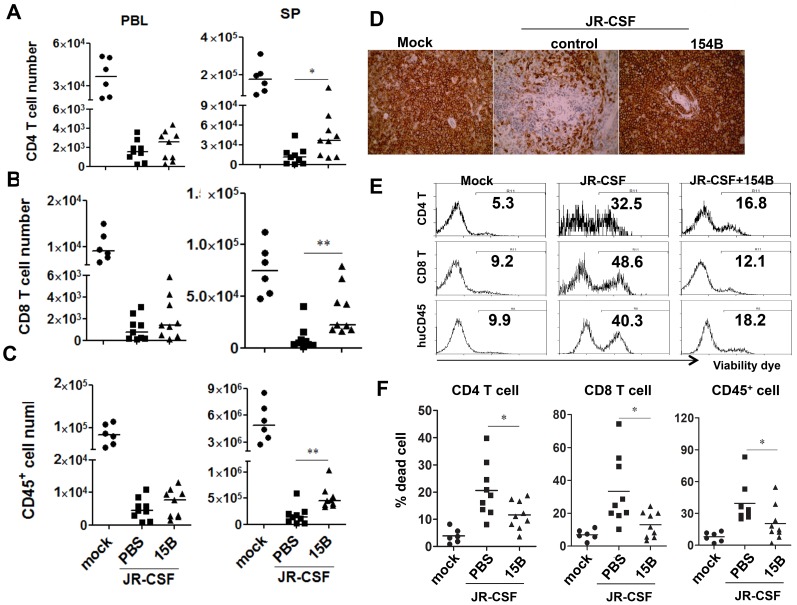
Depletion of pDC during chronic infection reduces HIV-1 induced immunopathogenesis. Humanized mice were infected HIV-JRCSF and were treated weekly with 15B or control at 11 weeks post-infection and terminated at 21 weeks post-infection (mock, *n* = 6; JR-CSF+control, *n* = 9; JR-CSF+15B, *n* = 9). (**A–C**) Summarized data show relative numbers in PBL and SP of (**A**) CD4 T cells (CD3^+^CD8^−^), (**B**) CD8 T cells (CD3^+^CD4^−^CD8^+^), and (**C**) human CD45^+^ leukocytes. (**D**) Immunohistochemistry staining for human CD45^+^ cells in spleens of mock or HIV-1 infected mice. (**E**) FACS plots of cell death in human CD4^+^ T cells, CD8^+^ T cells and huCD45^+^ leukocytes from spleens. (**F**) Summary data show percentages of cell death in CD4^+^ T cells, CD8^+^ T cells and huCD45^+^ leukocytes. Bars in dot graphs indicate median value. * and ** indicate p<0.05 and p<0.01, respectively.

In spite of the persistently higher viremia during 10 weeks of pDC depletion treatment, human CD4^+^ T cell numbers increased significantly in the spleen, comparing to the control group (Figure 7A, p<0.05). In addition, human CD8 T cells and CD45^+^ leukocyte numbers in spleens were also increased (Figure 7B&C, p<0.01). Interestingly, the relative depletion of human CD4, CD8 T cells and CD45 leukocytes was the same in the blood (Figure 7A–C). The increase of human CD45^+^ cells in the spleen was also confirmed by immunohistochemistry staining of spleen sections (Figure 7D). Accordingly, pDC depletion significantly reduced the percentage of dying cells in T cells and total human CD45^+^ cells in the spleen and other lymphoid organs (Figure 7E&F and data not shown). Therefore, persistent activation of pDC in lymphoid organs during HIV-1 chronic infection, although still producing IFN-I to suppress HIV-1 replication, contributes significantly to HIV-1 induced depletion of human leukocytes including human CD4 T cells.

## Discussion

HIV-1 infection induces a systemic immune activation [1], [47], which has been proposed to contribute to HIV-1 disease progression [1], [3], [4], [47], [48], [49]. Persistent activation of pDC and IFN-I have been correlated with HIV-1 infection induced immune activation both in HIV-infected patients and in SIV-infected rhesus monkeys [15], [20], [21],[16], [18], [23], [49], [50], [51]. The role of pDC in HIV-1 infection and immunopathogenesis, however, remains unclear [52], [53], [54]. Using the humanized mouse model of HIV-1 infection and pathogenesis in vivo, we developed a novel pDC-specific mAb that specifically and efficiently depletes human pDC in various lymphoid organs in vivo. We report here that, in response to acute HIV-1 infection, pDC are the critical IFN-I producer cells and contribute to suppressing HIV-1 replication during early HIV-1 infection. During chronic HIV-1 infection, pDC are still the major IFN-I producing cells and contribute to controlling HIV-1 replication. Most surprisingly, depletion of pDC during chronic HIV-1 infection rescued human leukocytes including human CD4 T cells in lymphoid organs but not in blood, in spite of higher levels of HIV-1 replication. We conclude that pDC play two opposing roles during HIV-1 infection and pathogenesis: they produce IFN-I to inhibit HIV-1 replication, but enhance HIV-1 pathogenesis by promoting cell death of human leukocytes including human CD4 and CD8 T cells.

Study of human pDC has been hampered by the difficulty of isolating sufficient number of human pDC cells and our inability to culture or expand pDC in vitro. Using humanized mice, we screened a number of human pDC-specific mAb and identified the 15B mAb that specifically and efficiently depletes human pDC in all lymphoid organs in vivo. With this mAb, we were able to define the role of pDC in HIV-1 replication and immunopathogenesis during different phases of HIV-1 infection. Our findings show that pDC are the critical or only IFN-I producer cells in response to acute HIV-1 infection in humanized mice. The pDC-independent production of IFN-α in plasma and ISG expression after pDC depletion during chronic HIV-1 infection may be due to the contribution of other cell types such as mDC and macrophages [55]. A recent report showed that TLR7 and TLR9 blockade had minimal impact on plasma IFN-α and expression of ISG in SIV-infected monkeys and did not alter viral load and T cell activation in vivo [56]. The discrepancy may be due to an incomplete blocking of pDC activation by the TLR7 and TLR9 antagonist in vivo or different contribution of other IFN-I producing cells in SIV-infected monkeys [56]. A recent report shows that pDC are the major IFN-I producing cells during primary SIV infection but the pDC pool is replenished by their precursors lacking IFN-I production capacity post acute phase infection [57]. However, we found that the depletion of pDC abolished or dramatically reduced IFN-I production during acute or chronic HIV-1 infection, suggesting that pDC are the major IFN-I producing cells during both acute and chronic HIV infection in humanized mice. The 15B mAb does not bind the BDCA2 receptor on monkey pDC (data not shown). The contribution of pDC in SIV-infected monkeys needs to be reexamined by pDC depletion when the appropriate depleting antibody is available.

The gut associated lymphoid tissue (GALT) is a major site of human T-cell depletion by HIV-1 infection. It is important to point out that the GALT in humanized mouse models are not structurally developed, with impaired intestinal lymphoid organ structure and human cell engulfment [58]. We thus can not examine the effect of pDC depletion in GALT in the current model. It will be interesting to examine the effect of pDC depletion on SIV pathogenesis in the GALT of SIV-infected monkeys, which recapitulates GALT pathogenesis as in HIV-infected humans.

HIV-1 disease progression is associated with gradual depletion of human leukocytes including other lineages as well as CD4 T cells [59], [60], [61]. Multiple mechanisms have been reported to account for the pathogenic activity of HIV-1 infection. Besides direct killing of HIV-1 infected CD4 cells, bystander cells including hematopoietic progenitor cells and uninfected human mature cells are also depleted, associated with immune hyperactivation during pathogenic HIV-1 infection. Several recent reports suggest that pDC may contribute to HIV-1 induced immune activation and subsequent immunopathogenesis. Repeated administrations of TLR7 ligands in mice induce AIDS-like lymphopenia, with reduced CD4^+^ T cells, CD8^+^ T cells and B cells [8]. It is reported that IFN-I triggers proapoptotic and antiproliferative effect on T cells [62].

We found that pDC-depletion not only rescued CD4 T cells but also CD8 T cells and total CD45^+^ leukocytes in lymphoid organs such as spleen and LN, but not in the blood. Therefore, persistent activation of pDC by HIV-1 infection in lymphoid organs contributes to HIV-1 immunopathogenesis by accelerating the death of all human leukocyte cells. Similarly, recent findings showed that treating SIV-infected rhesus macaques with the TLR7 and TLR9 antagonist DV056 led to a significant increase in levels of proliferating memory CD4 and CD8 T cells in the blood [56]. Interestingly, the anti-malaria drug chloroquine, which inhibits IFN-I production by pDC *in vitro* [63], appears to rescue human T cells in HIV-1 infected patients, correlated with reduced immune activation [10], [11]. However, two recent reports with similar chloroquine treatment failed to demonstrate the significant beneficial effect in HIV-1 patients [64], [65].

Two recent reports showed that blocking IFN-I signaling during LCMV persistent infection could improve antiviral T cell response and accelerate clearance of chronic LCMV infection via an IL-10-related mechanism [66], [67]. It is not clear if IFN-I also plays a similar critical role in HIV-1 infection and immunopathogenesis. It will be of great interest to test, when the human IFN-IR antagonistic mAb is available, if blocking human IFN-IR can similarly improve anti-HIV immunity and lead to better control of HIV-1 infection in humanized mice or in SIV-infected monkeys. However, pDC depletion in HIV-infected humanized mice showed very distinct outcomes as blocking IFN-I signaling in LCMV-infected mice. First, pDC depletion led to increased HIV-1 replication, and it did not affect IL-10 expression (Li, G. and Su, L., unpublished results). In addition, pDC depletion may remove IFN-I and additional factors expressed by “bad” pDC, including other inflammatory cytokines and cell death ligands such as TRAIL (killer pDC, [27], [28], [68]). Therefore, persistent pDC activation by HIV-1 infection contributes to HIV-1 induced depletion of human immune cells and leads to HIV-1 disease progression. Our findings suggest that depletion of “bad” pDC transiently during HIV-1 chronic infection may provide an effective treatment to preserve human immune cells in HIV-1 infected patients or in those HAART-treated immune non-responder patients [69], [70]. Recently, it was reported that blocking IFN-I with a non-signaling IFNα during acute SIV infection promoted viral replication, which is consistent with our data in the current study. However, they also observed accelerated CD4^+^ T-cell depletion and AIDS progression [71]. This may be due to the distinct experimental systems between pDC depletion in humanized mice and IFN-I blocking in SIV-infected monkeys. pDC have multiple functions beside IFN-I production, including production of other inflammatory cytokines, direct killing of T cells through TRAIL expression [68] and inhibition of antiviral immune response by Treg induction [18]. Therefore, pDC depletion in SIV-infected monkeys should be performed in future experiments to clarify the role of pDC in SIV infection and pathogenesis in various NHP hosts.

## Materials and Methods

### Ethics statement

The reports followed NIH research ethics guidelines. For the humanized mouse construction, human fetal liver were obtained from elective or medically indicated termination of pregnancy through a non-profit intermediary working with outpatient clinics (Advanced Bioscience Resources, Alameda, CA). The use of the tissue in research had no influence on the decision regarding termination of the pregnancy. Informed consent of the maternal donor is obtained in all cases, under regulation governing the clinic. We were provided with no information regarding the identity of the patients, nor is this information traceable. The project was reviewed by the University's Office of Human Research Ethics, which has determined that this submission does not constitute human subjects research as defined under federal regulations [45 CFR 46.102 (d or f) and 21 CFR 56.102(c)(e)(l)] and does not require IRB approval. The University of North Carolina at Chapel Hill Institutional Animal Care and Use Committee (IACUC) has reviewed and approved this research. All animal experiments were conducted following NIH guidelines for housing and care of laboratory animals and in accordance with The University of North Carolina at Chapel Hill in accordance with protocols approved by the institution's Institutional Animal Care and Use Committee (IACUC ID: 11-103.0).

### Construction of humanized mice

Approval for animal work was obtained from the University of North Carolina Institutional Animal Care and Use Committee (IACUC). We constructed Balb/C rag2-gammaC (DKO) mutant DKO-hu HSC or Nod-rag1-gammaC (NRG) NRG-hu HSC mice similarly as previously reported [39]. Briefly, human CD34^+^ cells were isolated from 16- to 20-week-old fetal liver tissues. Tissues were digested with Liver Digest Medium (Invitrogen, Frederick, MD). The suspension was filtered through a 70 µm cell strainer (BD Falcon, Lincoln Park, NJ) and was centrifuged at 150 g for 5 minutes to isolate mononuclear cells by Ficoll. After selection with the CD34^+^ magnetic-activated cell sorting (MACS) kit, CD34^+^ HSCs (0.5 x 10^6^) were injected into the liver of each 2- to 6-days old DKO or NRG mice, which had been previously irradiated at 300 rad. More than 95% of the humanized mice were stably reconstituted with human leukocytes in the blood (10%–90% at 12–14 weeks). Each cohort (Humanized mice reconstituted from the same human donor fetal liver tissue) had similar levels of engraftment. All mice were housed at the University of North Carolina at Chapel Hill.

### HIV-1 virus stocks and infection of humanized mice

NL4-R3A, generated by cloning a highly pathogenic dual tropic envelope into NL4-3 backbone [44], [45], [46], was used for acute infection experiment. An R5 tropic strain of HIV-1, JR-CSF, was used for both acute and chronic infection. All viruses were generated by transfection of 293T cells. Humanized mice with stable human leukocyte reconstitution were infected with NL4-R3A (5 ng p24/mouse) or JR-CSF (10 ng p24/mouse), through intravenous injection (*i.v.*). Humanized mice infected with 293T mock supernatant were used as control groups.

### Depletion of human pDC in humanized mice

A monoclonal antibody specific to blood dendritic cell antigen-2 (BDCA2), 15B, was used to treat humanized mice through intraperitoneal injection (i.p., 4 mg/kg). For acute HIV-1 infection, humanized mice were injected three times with 15B on -5, -3 and -1 days before infection. For acute R3A infection, mice will be treated on 3 and 6 days post-infection. For acute JR-CSF infection, mice will be treated every three days until termination. For chronic JR-CSF infection, 15B was applied to mice at 11wpi by injecting twice every week for 10 weeks.

### Flow cytometry

For HIV-1 gag p24 staining, cells were stained with surface markers first, and then permeabilized with cytofix/cytoperm buffer (BD Bioscience, cat#554714), followed by intracellular staining. Human leukocytes (mCD45^−^huCD45^+^) were analyzed for human CD3, CD4, CD8, CD123, HLA-DR and CD38 by CyAn FACS machine (Dako). FITC-conjugated anti–human HLA-DR (clone:L243, cat#307604), PE-conjugated anti-human CD38 (clone:HIT2, cat#303506), PE/Cy5-conjugated anti-human CD4 (clone:RP4-T4, cat#300510), PE/Cy7-conjugated anti-human CD3 (clone:HIT3a, cat#300316), Pacific blue-conjugated anti-human CD3 (clone:UCHT1, cat#300431), PE/Cy7-conjugated anti-human CD8 (clone:HIT8a, cat#300914), APC-conjugated human CD123 (clone:6H6, cat#306012) and APC/Cy7-conjuaged anti-human CD45 (clone:H130, cat#304014) were purchased from Biolegend; PE-conjugated anti-human caspase-3 (clone:C92-605, cat#51-68655X) was purchased from BD Bioscience. Pacific orange–conjugated anti–mouse CD45 (clone:HI30, cat#MHCD4530), PE/Texas red–conjugated anti–human CD4 (clone:S3.5, cat#MHCD0417) or CD8 (clone:3B5, cat#MHCD0817), and LIVE/DEAD Fixable Aqua Dead Cell Stain Kit (cat#L34957) were purchased from Invitrogen. FITC-conjugated anti-HIV p24 (clone:FH190-1-1, cat#6604665) was purchased from Beckman Coulter. The cells were analyzed on a CyAn ADP (Dako).

### Immunohistochemistry

Paraffin-embedded spleen sections from humanized mice were stained with the mouse anti–human CD45 (Dako, cat#N1514) or HIV-1 p24 antibody (Dako, cat#M0857), washed in PBS, then incubated with Mouse-&-Rabbit-on-Rodent Double Stain Polymer (BIOCARE MEDICAL, cat#RDS513H) and substrate DAB (BIOCARE MEDICAL, cat#BDB2004 H, L, MM). Images were captured using a QImaging Micropublisher 3.3 CCD digital camera and QCapture software version 3.0 (QImaging, Surrey, BC).

### Cellular mRNA level detection

Interferon alpha-1/13 (IFNα1/13), interferon alpha-2 (IFNα2), interferon beta (IFNβ) [72], interferon gamma (IFNγ) [73] and tumor necrosis factor alpha (TNFα) [74] were detected. IFN-I stimulated genes, MxA [75] and TRIM22 [76], were detected to confirm pDCs depletion effect on type I IFN production. Real-time PCR assay was performed (ABI Applied Biosystem). All samples were tested in triplicate using the human GAPDH gene [77] for normalization.

### Statistical analysis

Data were analyzed using GraphPad Prism software version 5.0 (GraphPad software, San Diego, CA, USA). The methods used for analysis of microarray data were described above. The data from different infection groups of mice were compared using a 2-tailed Mann-Whitney U test. For gene expression, mean-ΔCT was calculated as the average (± SD) of all ΔCT values within each group of samples and 2-way ANOVA method was used. Correlations were estimated with a Spearman test. All results were considered significant when p<0.05.

## Supporting Information

Figure S1 (**A–E**) Kinetics of HIV-JRCSF infection and immunopathogenesis in humanized mice measured by quantitative real-time PCR (*n* = 10, **A**), IFNa2 induction (**B**), immune activation of human CD8 T cells (**C**), relative percentages of CD4 T cells in the blood (PBL) or spleens (**D**), or total cell numbers of CD4, CD8 T cells and human CD45+ leukocytes (**E**). (C–E) All mice were analyzed at 18 weeks post HIV infection. Each dot represents one mouse and ** indicate p<0.01.
(TIF)

Figure S2 Specific depletion of pDCs induced by 15B in different lymphoid organs in humanized mice. Representative FACS plots show percentages of CD3+CD19- T cells and CD3-CD19+ B cells in huCD45+ cells (**A**) or CD3-CD11c+ mDC (**B**) in the blood, mLN and spleens.
(TIF)

Figure S3 Specific depletion of pDCs induced by 15B in different lymphoid organs in humanized mice. (**A**) Representative FACS plots and summarized data (**B**) show percentages of CD3-CD14+ cell in huCD45+ cells in the blood, mLN and spleens.
(TIF)

Figure S4 Relative T-cell activation in humanized mice with or without pDC depletion. (A) pDC were depleted before HIV infection, the percentage of HLA-DR^+^CD38^+^ of CD8 T cells in the spleen at 8 days post-infection by R3A is summarized. (B) pDC were depleted before HIV infection, the percentage of HLA-DR^+^CD38^+^ of CD8 T cells in the spleen at 3 weeks post-infection by JR-CSF is summarized. * indicates p<0.05.
(TIF)

Figure S5 Depletion of pDC during chronic HIV-1 infection reduces type I IFN response. Humanized mice were infected with HIV-JRCSF and treated with 15B or control at 11 weeks post-infection and terminated at 21 weeks post-infection. Human cells (CD45+ or CD3+ CD8+ T cells) from spleens of mock, HIV-1/control or HIV-1/15B mice were purified by flow cytometry. Total mRNA were isolated and used for the cDNA microarray assay. Gene expression of a panel of ISGs relative to mock samples in human CD45+ cells (left) and CD3+CD4-CD8+T cells (right) is shown. The relative expression over mock samples is indicated by the color bars.
(TIF)

## References

[1] Ascher MS, Sheppard HW (1988) AIDS as immune system activation: a model for pathogenesis. Clin Exp Immunol 73: 165–167. 3263225 PMC1541594

[2] Sodora DL, Silvestri G (2008) Immune activation and AIDS pathogenesis. Aids 22: 439–446. 18301056 10.1097/QAD.0b013e3282f2dbe7

[3] Moir S, Chun TW, Fauci AS (2011) Pathogenic mechanisms of HIV disease. Annu Rev Pathol 6: 223–248. 21034222 10.1146/annurev-pathol-011110-130254

[4] Giorgi JV, Liu Z, Hultin LE, Cumberland WG, Hennessey K, et al (1993) Elevated levels of CD38+ CD8+ T cells in HIV infection add to the prognostic value of low CD4+ T cell levels: results of 6 years of follow-up. The Los Angeles Center, Multicenter AIDS Cohort Study. J Acquir Immune Defic Syndr 6: 904–912. 7686224

[5] Apetrei C, Sumpter B, Souquiere S, Chahroudi A, Makuwa M, et al (2011) Immunovirological analyses of chronically simian immunodeficiency virus SIVmnd-1- and SIVmnd-2-infected mandrills (Mandrillus sphinx). J Virol 85: 13077–13087. 21957286 10.1128/JVI.05693-11 PMC3233116

[6] Klatt NR, Estes JD, Sun X, Ortiz AM, Barber JS, et al (2012) Loss of mucosal CD103+ DCs and IL-17+ and IL-22+ lymphocytes is associated with mucosal damage in SIV infection. Mucosal Immunol 5: 646–657. 22643849 10.1038/mi.2012.38 PMC3443541

[7] Heikenwalder M, Polymenidou M, Junt T, Sigurdson C, Wagner H, et al (2004) Lymphoid follicle destruction and immunosuppression after repeated CpG oligodeoxynucleotide administration. Nat Med 10: 187–192. 14745443 10.1038/nm987

[8] Baenziger S, Heikenwalder M, Johansen P, Schlaepfer E, Hofer U, et al (2009) Triggering TLR7 in mice induces immune activation and lymphoid system disruption, resembling HIV-mediated pathology. Blood 113: 377–388. 18824599 10.1182/blood-2008-04-151712

[9] Pandrea I, Sodora DL, Silvestri G, Apetrei C (2008) Into the wild: simian immunodeficiency virus (SIV) infection in natural hosts. Trends Immunol 29: 419–428. 18676179 10.1016/j.it.2008.05.004 PMC2840226

[10] Murray SM, Down CM, Boulware DR, Stauffer WM, Cavert WP, et al (2010) Reduction of immune activation with chloroquine therapy during chronic HIV infection. J Virol 84: 12082–12086. 20844049 10.1128/JVI.01466-10 PMC2977889

[11] Piconi S, Parisotto S, Rizzardini G, Passerini S, Terzi R, et al (2011) Hydroxychloroquine drastically reduces immune activation in HIV-infected, antiretroviral therapy-treated immunologic nonresponders. Blood 118: 3263–3272. 21576701 10.1182/blood-2011-01-329060

[12] Brenchley JM, Price DA, Schacker TW, Asher TE, Silvestri G, et al (2006) Microbial translocation is a cause of systemic immune activation in chronic HIV infection. Nat Med 12: 1365–1371. 17115046 10.1038/nm1511

[13] Fitzgerald-Bocarsly P, Jacobs ES (2010) Plasmacytoid dendritic cells in HIV infection: striking a delicate balance. J Leukoc Biol 87: 609–620. 20145197 10.1189/jlb.0909635 PMC2858309

[14] Skurkovich S, Skurkovich B, Bellanti JA (1993) A disturbance of interferon synthesis with the hyperproduction of unusual kinds of interferon can trigger autoimmune disease and play a pathogenetic role in AIDS: the removal of these interferons can be therapeutic. Med Hypotheses 41: 177–185. 7694057 10.1016/0306-9877(93)90066-y

[15] Meier A, Chang JJ, Chan ES, Pollard RB, Sidhu HK, et al (2009) Sex differences in the Toll-like receptor-mediated response of plasmacytoid dendritic cells to HIV-1. Nat Med 15: 955–959. 19597505 10.1038/nm.2004 PMC2821111

[16] Campillo-Gimenez L, Laforge M, Fay M, Brussel A, Cumont MC, et al (2010) Nonpathogenesis of simian immunodeficiency virus infection is associated with reduced inflammation and recruitment of plasmacytoid dendritic cells to lymph nodes, not to lack of an interferon type I response, during the acute phase. J Virol 84: 1838–1846. 19939930 10.1128/JVI.01496-09 PMC2812402

[17] Jacquelin B, Mayau V, Targat B, Liovat AS, Kunkel D, et al (2009) Nonpathogenic SIV infection of African green monkeys induces a strong but rapidly controlled type I IFN response. J Clin Invest 119: 3544–3555. 19959873 10.1172/JCI40093 PMC2786805

[18] Manches O, Munn D, Fallahi A, Lifson J, Chaperot L, et al (2008) HIV-activated human plasmacytoid DCs induce Tregs through an indoleamine 2,3-dioxygenase-dependent mechanism. J Clin Invest 118: 3431–3439. 18776940 10.1172/JCI34823 PMC2528911

[19] Baca-Regen L, Heinzinger N, Stevenson M, Gendelman HE (1994) Alpha interferon-induced antiretroviral activities: restriction of viral nucleic acid synthesis and progeny virion production in human immunodeficiency virus type 1-infected monocytes. J Virol 68: 7559–7565. 7933143 10.1128/jvi.68.11.7559-7565.1994 PMC237202

[20] Buimovici-Klein E, Lange M, Klein RJ, Cooper LZ, Grieco MH (1983) Is presence of interferon predictive for AIDS? Lancet 2: 344. 10.1016/s0140-6736(83)90322-7 6135859

[21] Buimovici-Klein E, Lange M, Klein RJ, Grieco MH, Cooper LZ (1986) Long-term follow-up of serum-interferon and its acid-stability in a group of homosexual men. AIDS Res 2: 99–108. 3487332 10.1089/aid.1.1986.2.99

[22] Bosinger SE, Li Q, Gordon SN, Klatt NR, Duan L, et al (2009) Global genomic analysis reveals rapid control of a robust innate response in SIV-infected sooty mangabeys. J Clin Invest 119: 3556–3572. 19959874 10.1172/JCI40115 PMC2786806

[23] Harris LD, Tabb B, Sodora DL, Paiardini M, Klatt NR, et al (2010) Downregulation of robust acute type I interferon responses distinguishes nonpathogenic simian immunodeficiency virus (SIV) infection of natural hosts from pathogenic SIV infection of rhesus macaques. J Virol 84: 7886–7891. 20484518 10.1128/JVI.02612-09 PMC2897601

[24] Liu YJ (2005) IPC: professional type 1 interferon-producing cells and plasmacytoid dendritic cell precursors. Annu Rev Immunol 23: 275–306. 15771572 10.1146/annurev.immunol.23.021704.115633

[25] Nascimbeni M, Perie L, Chorro L, Diocou S, Kreitmann L, et al (2009) Plasmacytoid dendritic cells accumulate in spleens from chronically HIV-infected patients but barely participate in interferon-alpha expression. Blood 113: 6112–6119. 19366987 10.1182/blood-2008-07-170803

[26] Herbeuval JP, Hardy AW, Boasso A, Anderson SA, Dolan MJ, et al (2005) Regulation of TNF-related apoptosis-inducing ligand on primary CD4+ T cells by HIV-1: role of type I IFN-producing plasmacytoid dendritic cells. Proc Natl Acad Sci U S A 102: 13974–13979. 16174727 10.1073/pnas.0505251102 PMC1224361

[27] Herbeuval JP, Nilsson J, Boasso A, Hardy AW, Kruhlak MJ, et al (2006) Differential expression of IFN-alpha and TRAIL/DR5 in lymphoid tissue of progressor versus nonprogressor HIV-1-infected patients. Proc Natl Acad Sci U S A 103: 7000–7005. 16632604 10.1073/pnas.0600363103 PMC1444883

[28] Stary G, Klein I, Kohlhofer S, Koszik F, Scherzer T, et al (2009) Plasmacytoid dendritic cells express TRAIL and induce CD4+ T-cell apoptosis in HIV-1 viremic patients. Blood 114: 3854–3863. 19690337 10.1182/blood-2009-04-217927

[29] Chehimi J, Papasavvas E, Tomescu C, Gekonge B, Abdulhaqq S, et al (2010) Inability of plasmacytoid dendritic cells to directly lyse HIV-infected autologous CD4+ T cells despite induction of tumor necrosis factor-related apoptosis-inducing ligand. J Virol 84: 2762–2773. 20042498 10.1128/JVI.01350-09 PMC2826047

[30] Donaghy H, Pozniak A, Gazzard B, Qazi N, Gilmour J, et al (2001) Loss of blood CD11c(+) myeloid and CD11c(−) plasmacytoid dendritic cells in patients with HIV-1 infection correlates with HIV-1 RNA virus load. Blood 98: 2574–2576. 11588058 10.1182/blood.v98.8.2574

[31] Feldman S, Stein D, Amrute S, Denny T, Garcia Z, et al (2001) Decreased interferon-alpha production in HIV-infected patients correlates with numerical and functional deficiencies in circulating type 2 dendritic cell precursors. Clin Immunol 101: 201–210. 11683579 10.1006/clim.2001.5111

[32] Pacanowski J, Kahi S, Baillet M, Lebon P, Deveau C, et al (2001) Reduced blood CD123+ (lymphoid) and CD11c+ (myeloid) dendritic cell numbers in primary HIV-1 infection. Blood 98: 3016–3021. 11698285 10.1182/blood.v98.10.3016

[33] Soumelis V, Scott I, Gheyas F, Bouhour D, Cozon G, et al (2001) Depletion of circulating natural type 1 interferon-producing cells in HIV-infected AIDS patients. Blood 98: 906–912. 11493432 10.1182/blood.v98.4.906

[34] Siegal FP, Lopez C, Fitzgerald PA, Shah K, Baron P, et al (1986) Opportunistic infections in acquired immune deficiency syndrome result from synergistic defects of both the natural and adaptive components of cellular immunity. J Clin Invest 78: 115–123. 3088039 10.1172/JCI112539 PMC329539

[35] Lichtner M, Rossi R, Rizza MC, Mengoni F, Sauzullo I, et al (2008) Plasmacytoid dendritic cells count in antiretroviral-treated patients is predictive of HIV load control independent of CD4+ T-cell count. Curr HIV Res 6: 19–27. 18288971 10.2174/157016208783571937

[36] McCune J, Kaneshima H, Krowka J, Namikawa R, Outzen H, et al (1991) The SCID-hu mouse: a small animal model for HIV infection and pathogenesis. Annu Rev Immunol 9: 399–429. 1910684 10.1146/annurev.iy.09.040191.002151

[37] Baenziger S, Tussiwand R, Schlaepfer E, Mazzucchelli L, Heikenwalder M, et al (2006) Disseminated and sustained HIV infection in CD34+ cord blood cell-transplanted Rag2-/-gamma c-/- mice. Proc Natl Acad Sci U S A 103: 15951–15956. 17038503 10.1073/pnas.0604493103 PMC1635108

[38] Berges BK, Wheat WH, Palmer BE, Connick E, Akkina R (2006) HIV-1 infection and CD4 T cell depletion in the humanized Rag2-/-gamma c-/- (RAG-hu) mouse model. Retrovirology 3: 76. 17078891 10.1186/1742-4690-3-76 PMC1635423

[39] Zhang L, Kovalev GI, Su L (2007) HIV-1 infection and pathogenesis in a novel humanized mouse model. Blood 109: 2978–2981. 17132723 10.1182/blood-2006-07-033159 PMC1852218

[40] Sun Z, Denton PW, Estes JD, Othieno FA, Wei BL, et al (2007) Intrarectal transmission, systemic infection, and CD4+ T cell depletion in humanized mice infected with HIV-1. J Exp Med 204: 705–714. 17389241 10.1084/jem.20062411 PMC2118553

[41] Zhang L, Jiang Q, Li G, Jeffrey J, Kovalev GI, et al (2011) Efficient infection, activation, and impairment of pDCs in the BM and peripheral lymphoid organs during early HIV-1 infection in humanized rag2(-)/(-)gamma C(-)/(-) mice in vivo. Blood 117: 6184–6192. 21505190 10.1182/blood-2011-01-331173 PMC3122941

[42] Traggiai E, Chicha L, Mazzucchelli L, Bronz L, Piffaretti JC, et al (2004) Development of a human adaptive immune system in cord blood cell-transplanted mice. Science 304: 104–107. 15064419 10.1126/science.1093933

[43] Tanaka S, Saito Y, Kunisawa J, Kurashima Y, Wake T, et al (2012) Development of Mature and Functional Human Myeloid Subsets in Hematopoietic Stem Cell-Engrafted NOD/SCID/IL2rgammaKO Mice. J Immunol 188 (12) 6145–55. 22611244 10.4049/jimmunol.1103660 PMC3370073

[44] Meissner EG, Duus KM, Gao F, Yu XF, Su L (2004) Characterization of a thymus-tropic HIV-1 isolate from a rapid progressor: role of the envelope. Virology 328: 74–88. 15380360 10.1016/j.virol.2004.07.019 PMC4429060

[45] Sivaraman V, Zhang L, Meissner EG, Jeffrey JL, Su L (2009) The heptad repeat 2 domain is a major determinant for enhanced human immunodeficiency virus type 1 (HIV-1) fusion and pathogenicity of a highly pathogenic HIV-1 Env. J Virol 83: 11715–11725. 19726524 10.1128/JVI.00649-09 PMC2772666

[46] Meissner EG, Coffield VM, Su L (2005) Thymic pathogenicity of an HIV-1 envelope is associated with increased CXCR4 binding efficiency and V5-gp41-dependent activity, but not V1/V2-associated CD4 binding efficiency and viral entry. Virology 336: 184–197. 15892960 10.1016/j.virol.2005.03.032 PMC4415377

[47] Lane HC, Masur H, Edgar LC, Whalen G, Rook AH, et al (1983) Abnormalities of B-cell activation and immunoregulation in patients with the acquired immunodeficiency syndrome. N Engl J Med 309: 453–458. 6224088 10.1056/NEJM198308253090803

[48] Grossman Z, Bentwich Z, Herberman RB (1993) From HIV infection to AIDS: are the manifestations of effective immune resistance misinterpreted? Clin Immunol Immunopathol 69: 123–135. 8403549 10.1006/clin.1993.1160

[49] Bosinger SE, Sodora DL, Silvestri G (2011) Generalized immune activation and innate immune responses in simian immunodeficiency virus infection. Curr Opin HIV AIDS 6: 411–418. 21743324 10.1097/COH.0b013e3283499cf6 PMC3261611

[50] Kwa S, Kannanganat S, Nigam P, Siddiqui M, Shetty RD, et al (2011) Plasmacytoid dendritic cells are recruited to the colorectum and contribute to immune activation during pathogenic SIV infection in rhesus macaques. Blood 118: 2763–2773. 21693759 10.1182/blood-2011-02-339515 PMC3172794

[51] Manches O, Fernandez MV, Plumas J, Chaperot L, Bhardwaj N (2012) Activation of the noncanonical NF-kappaB pathway by HIV controls a dendritic cell immunoregulatory phenotype. Proc Natl Acad Sci U S A 109: 14122–14127. 22879398 10.1073/pnas.1204032109 PMC3435221

[52] Riviere Y, Gresser I, Guillon JC, Bandu MT, Ronco P, et al (1980) Severity of lymphocytic choriomeningitis virus disease in different strains of suckling mice correlates with increasing amounts of endogenous interferon. J Exp Med 152: 633–640. 6157772 10.1084/jem.152.3.633 PMC2185917

[53] Wang Y, Swiecki M, Cella M, Alber G, Schreiber RD, et al (2012) Timing and magnitude of type I interferon responses by distinct sensors impact CD8 T cell exhaustion and chronic viral infection. Cell Host Microbe 11: 631–642. 22704623 10.1016/j.chom.2012.05.003 PMC3572910

[54] Cervantes-Barragan L, Lewis KL, Firner S, Thiel V, Hugues S, et al (2012) Plasmacytoid dendritic cells control T-cell response to chronic viral infection. Proc Natl Acad Sci U S A 109: 3012–3017. 22315415 10.1073/pnas.1117359109 PMC3286988

[55] Lepelley A, Louis S, Sourisseau M, Law HK, Pothlichet J, et al (2011) Innate sensing of HIV-infected cells. PLoS Pathog 7: e1001284. 21379343 10.1371/journal.ppat.1001284 PMC3040675

[56] Kader M, Smith AP, Guiducci C, Wonderlich ER, Normolle D, et al (2013) Blocking TLR7- and TLR9-mediated IFN-alpha production by plasmacytoid dendritic cells does not diminish immune activation in early SIV infection. PLoS Pathog 9: e1003530. 23935491 10.1371/journal.ppat.1003530 PMC3723633

[57] Bruel T, Dupuy S, Demoulins T, Rogez-Kreuz C, Dutrieux J, et al (2014) Plasmacytoid dendritic cell dynamics tune interferon-alfa production in SIV-infected cynomolgus macaques. PLoS Pathog 10: e1003915. 24497833 10.1371/journal.ppat.1003915 PMC3907389

[58] Hofer U, Baenziger S, Heikenwalder M, Schlaepfer E, Gehre N, et al (2008) RAG2-/- gamma(c)-/- mice transplanted with CD34+ cells from human cord blood show low levels of intestinal engraftment and are resistant to rectal transmission of human immunodeficiency virus. J Virol 82: 12145–12153. 18842716 10.1128/JVI.01105-08 PMC2593344

[59] Margolick JB, Munoz A, Donnenberg AD, Park LP, Galai N, et al (1995) Failure of T-cell homeostasis preceding AIDS in HIV-1 infection. The Multicenter AIDS Cohort Study. Nat Med 1: 674–680. 7585150 10.1038/nm0795-674

[60] Scadden DT, Shen H, Cheng T (2001) Hematopoietic stem cells in HIV disease. J Natl Cancer Inst Monogr 24–29. 11158203 10.1093/oxfordjournals.jncimonographs.a024253

[61] Suzu S, Harada H, Matsumoto T, Okada S (2005) HIV-1 Nef interferes with M-CSF receptor signaling through Hck activation and inhibits M-CSF bioactivities. Blood 105: 3230–3237. 15626739 10.1182/blood-2004-06-2084

[62] Tanabe Y, Nishibori T, Su L, Arduini RM, Baker DP, et al (2005) Cutting edge: role of STAT1, STAT3, and STAT5 in IFN-alpha beta responses in T lymphocytes. J Immunol 174: 609–613. 15634877 10.4049/jimmunol.174.2.609

[63] Beignon AS, McKenna K, Skoberne M, Manches O, DaSilva I, et al (2005) Endocytosis of HIV-1 activates plasmacytoid dendritic cells via Toll-like receptor-viral RNA interactions. J Clin Invest 115: 3265–3275. 16224540 10.1172/JCI26032 PMC1253628

[64] Vaccari M, Fenizia C, Ma ZM, Hryniewicz A, Boasso A, et al (2014) Transient increase of interferon-stimulated genes and no clinical benefit by chloroquine treatment during acute simian immunodeficiency virus infection of macaques. AIDS Res Hum Retroviruses 30: 355–362. 24251542 10.1089/aid.2013.0218 PMC3976588

[65] Paton NI, Goodall RL, Dunn DT, Franzen S, Collaco-Moraes Y, et al (2012) Effects of hydroxychloroquine on immune activation and disease progression among HIV-infected patients not receiving antiretroviral therapy: a randomized controlled trial. JAMA 308: 353–361. 22820788 10.1001/jama.2012.6936 PMC3821003

[66] Wilson EB, Yamada DH, Elsaesser H, Herskovitz J, Deng J, et al (2013) Blockade of chronic type I interferon signaling to control persistent LCMV infection. Science 340: 202–207. 23580528 10.1126/science.1235208 PMC3704950

[67] Teijaro JR, Ng C, Lee AM, Sullivan BM, Sheehan KC, et al (2013) Persistent LCMV infection is controlled by blockade of type I interferon signaling. Science 340: 207–211. 23580529 10.1126/science.1235214 PMC3640797

[68] Hardy AW, Graham DR, Shearer GM, Herbeuval JP (2007) HIV turns plasmacytoid dendritic cells (pDC) into TRAIL-expressing killer pDC and down-regulates HIV coreceptors by Toll-like receptor 7-induced IFN-alpha. Proc Natl Acad Sci U S A 104: 17453–17458. 17956986 10.1073/pnas.0707244104 PMC2077277

[69] Sauce D, Larsen M, Fastenackels S, Pauchard M, Ait-Mohand H, et al (2011) HIV disease progression despite suppression of viral replication is associated with exhaustion of lymphopoiesis. Blood 117: 5142–5151. 21436070 10.1182/blood-2011-01-331306 PMC3109539

[70] Zhang Z, Fu J, Xu X, Wang S, Xu R, et al (2013) Safety and immunological responses to human mesenchymal stem cell therapy in difficult-to-treat HIV-1-infected patients. AIDS 27: 1283–1293. 23925377 10.1097/QAD.0b013e32835fab77 PMC4329727

[71] Douek DC (2014) Perturbing Interferon Signaling in SIV Infection. Conference on Retroviruses and Opportunistic Infections. Boston, MA.

[72] Puig M, Tosh KW, Schramm LM, Grajkowska LT, Kirschman KD, et al (2012) TLR9 and TLR7 agonists mediate distinct type I IFN responses in humans and nonhuman primates in vitro and in vivo. J Leukoc Biol 91: 147–158. 22058422 10.1189/jlb.0711371

[73] Poloni A, Sartini D, Emanuelli M, Trappolini S, Mancini S, et al (2011) Gene expression profile of cytokines in patients with chronic graft-versus-host disease after allogeneic hematopoietic stem cell transplantation with reduced conditioning. Cytokine 53: 376–383. 21211989 10.1016/j.cyto.2010.12.008

[74] Liu X, Silverstein PS, Singh V, Shah A, Qureshi N, et al (2012) Methamphetamine increases LPS-mediated expression of IL-8, TNF-alpha and IL-1beta in human macrophages through common signaling pathways. PLoS One 7: e33822. 22479453 10.1371/journal.pone.0033822 PMC3315580

[75] Urosevic M, Dummer R, Conrad C, Beyeler M, Laine E, et al (2005) Disease-independent skin recruitment and activation of plasmacytoid predendritic cells following imiquimod treatment. J Natl Cancer Inst 97: 1143–1153. 16077073 10.1093/jnci/dji207

[76] Singh R, Gaiha G, Werner L, McKim K, Mlisana K, et al (2011) Association of TRIM22 with the type 1 interferon response and viral control during primary HIV-1 infection. J Virol 85: 208–216. 20980524 10.1128/JVI.01810-10 PMC3014203

[77] Washburn ML, Bility MT, Zhang L, Kovalev GI, Buntzman A, et al (2011) A humanized mouse model to study hepatitis C virus infection, immune response, and liver disease. Gastroenterology 140: 1334–1344. 21237170 10.1053/j.gastro.2011.01.001 PMC3066273

