# Supplementary figures and images for: Plasmacytoid Dendritic Cells Suppress HIV-1 Replication but Contribute to HIV-1 Induced Immunopathogenesis in Humanized Mice
Source: PLoS Pathog. 2014 Jul 31;10(7):e1004291. doi: 10.1371/journal.ppat.1004291 (PMC4117636; doi:10.1371/journal.ppat.1004291)

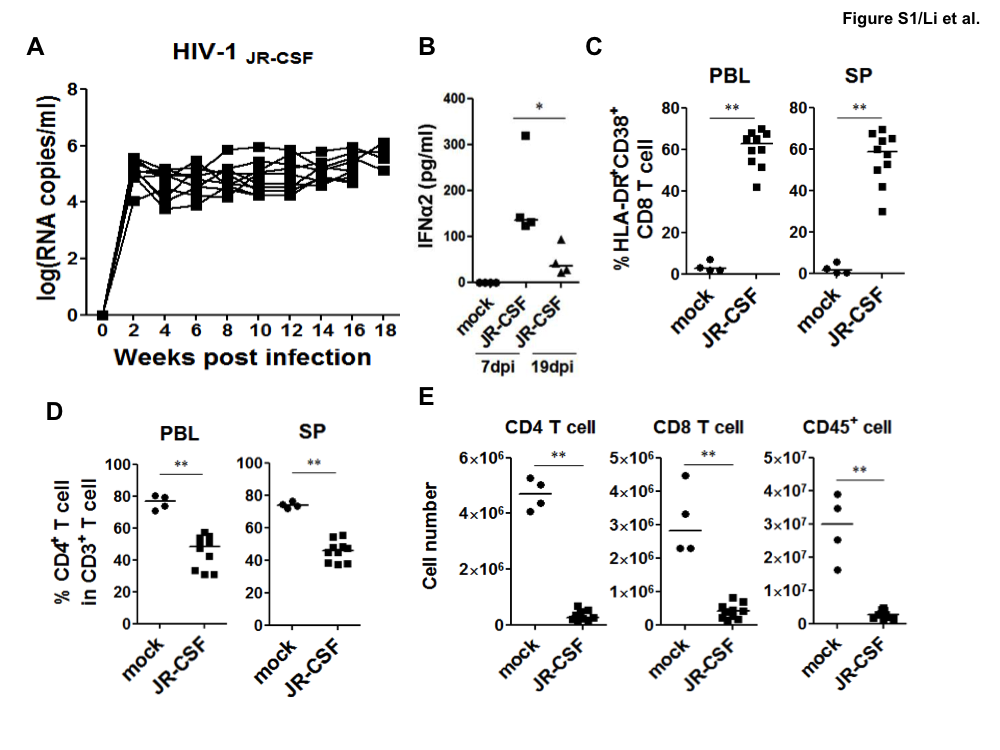

Supplement: Figure S1 — (A–E) Kinetics of HIV-JRCSF infection and immunopathogenesis in humanized mice measured by quantitative real-time PCR (n = 10, A), IFNa2 induction (B), immune activation of human CD8 T cells (C), relative percentages of CD4 T cells in the blood (PBL) or spleens (D), or total cell numbers of CD4, CD8 T cells and human CD45+ leukocytes (E). (C–E) All mice were analyzed at 18 weeks post HIV infection. Each dot represents one mouse and ** indicate p<0.01. (TIF) [file ppat.1004291.s001.tif]

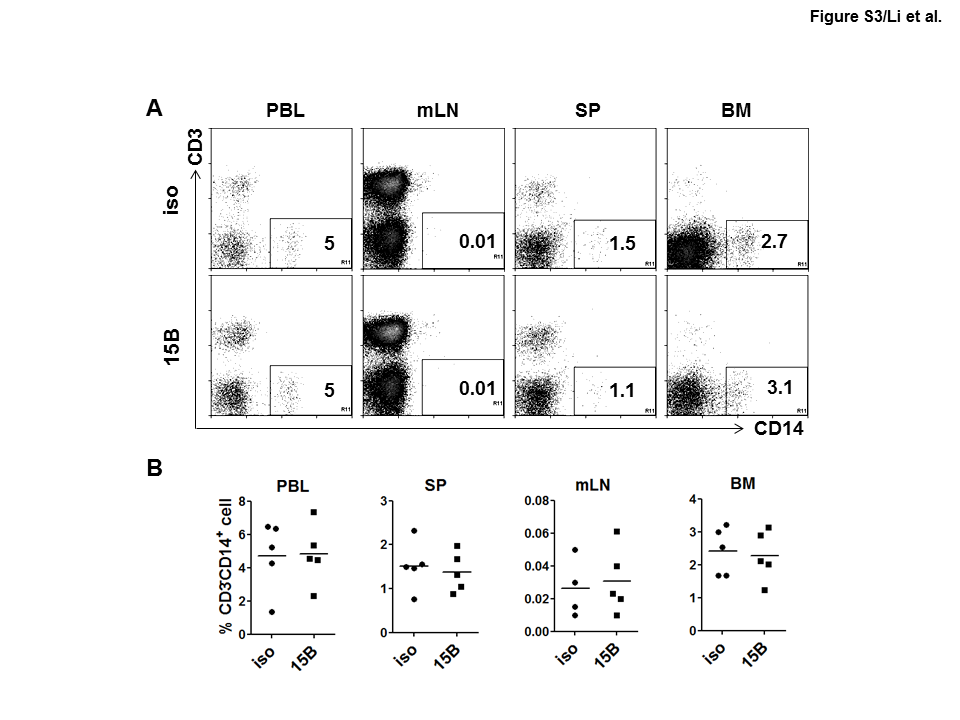

Supplement: Figure S3 — Specific depletion of pDCs induced by 15B in different lymphoid organs in humanized mice. (A) Representative FACS plots and summarized data (B) show percentages of CD3-CD14+ cell in huCD45+ cells in the blood, mLN and spleens. (TIF) [file ppat.1004291.s003.tif]

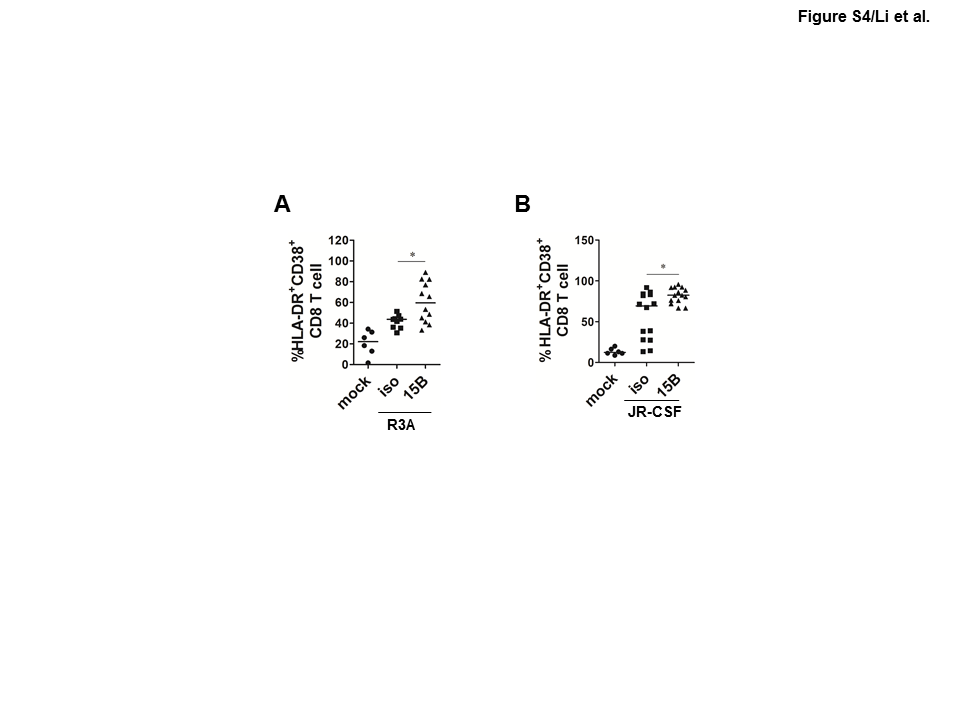

Supplement: Figure S4 — Relative T-cell activation in humanized mice with or without pDC depletion. (A) pDC were depleted before HIV infection, the percentage of HLA-DR+CD38+ of CD8 T cells in the spleen at 8 days post-infection by R3A is summarized. (B) pDC were depleted before HIV infection, the percentage of HLA-DR+CD38+ of CD8 T cells in the spleen at 3 weeks post-infection by JR-CSF is summarized. * indicates p<0.05. (TIF) [file ppat.1004291.s004.tif]

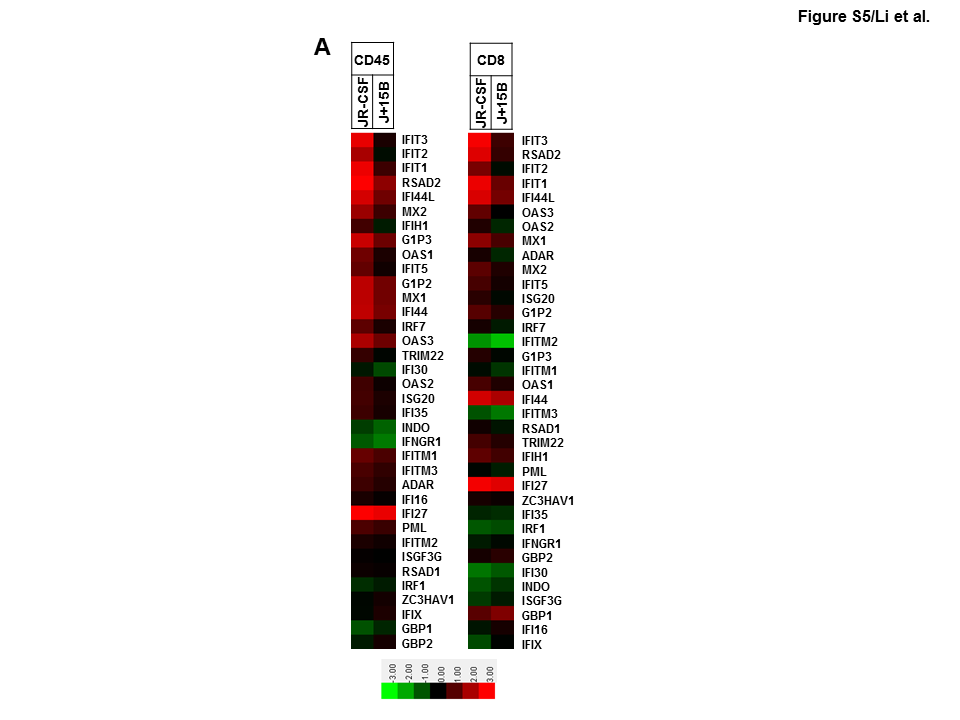

Supplement: Figure S5 — Depletion of pDC during chronic HIV-1 infection reduces type I IFN response. Humanized mice were infected with HIV-JRCSF and treated with 15B or control at 11 weeks post-infection and terminated at 21 weeks post-infection. Human cells (CD45+ or CD3+ CD8+ T cells) from spleens of mock, HIV-1/control or HIV-1/15B mice were purified by flow cytometry. Total mRNA were isolated and used for the cDNA microarray assay. Gene expression of a panel of ISGs relative to mock samples in human CD45+ cells (left) and CD3+CD4-CD8+T cells (right) is shown. The relative expression over mock samples is indicated by the color bars. (TIF) [file ppat.1004291.s005.tif]
